# Personal control of privacy and data: Estonian experience

**DOI:** 10.1007/s12553-017-0195-1

**Published:** 2017-06-15

**Authors:** Jaan Priisalu, Rain Ottis

**Affiliations:** 1 Department of Software Science, School of Information Technologies, TUT Centre for Digital Forensics and Cyber Security, Tallinn University of Technology, Tallinn, Estonia; 2 NATO Cooperative Cyber Defence Centre of Excellence, Tallinn, Estonia

**Keywords:** Privacy, E-government, Estonia, Information security, Integrity

## Abstract

The Republic of Estonia leads Europe in the provision of public digital services. The national communications and transactions platform allows for twenty-first century governance by allowing for transparency, e-safety (inter alia privacy), e-security, entrepreneurship and, among other things, rising levels of prosperity, and well-being for all its Citizens. However, a series of Information Infrastructure attacks against the Estonian e-society infrastructure in 2007 became one of best known incidents and experiences that fundamentally changed both Estonian and international discussions about Cyber Security and Privacy. Estonian experience shows that an open and transparent attitude provides a good foundation for trust between the Citizen and the State, and gives more control to the real owner of the data - the Citizen. Another important lesson is that the Citizen needs to be confident in the government’s ability to keep their data safe -- in terms of confidentiality, integrity and availability - establishing a strong link between privacy and information security. This paper discusses certain critical choices, context, and events connected to the birth and growth of the Estonian e-society in terms of Privacy.

## Introduction

According to the Digital Economy and Society index of 2017, the Republic of Estonia “is the champion in Europe in the online provision of public services” [6], businesses and the state can take care of most of their interactions over the Internet. The government’s intention to create a modern and thriving e-society infrastructure, in order to support the present and future public and private sectors, has encountered many challenges and great strife. Nevertheless, the government of Estonia has moved forward to address those challenges head-on, with an innovative development and deployment plan^1^ [14]. The intention at the time of its inception was that an Estonian e-society infrastructure will allow for a twenty-first century means of governance to exist, by allowing for transparency, e-safety, e-security,^2^ entrepreneurship and, inter alia, rising levels of prosperity and well-being for all citizens. All of these goals are closely linked with the Privacy of the citizens whose data is processed by the government.

Privacy is a complicated concept that has various definitions [24]. The European Court of Human Rights has ruled that the concept of “private life” does not have an “exhaustive definition” [15, 2] and as such there is no universal definition to which we can refer to. Furthermore, this paper only focuses on the aspect of Privacy that deals with Citizen data that is stored and processed in Estonian Government information systems. In order to better understand the meaning of privacy in this context, we turn to the laws of the Estonian Republic. §26 of the Estonian Constitution [25] states the right to private life^3^ and further states that the government (officials) can only breach this right, in situations enumerated by law. §43 establishes the right to message secrecy^4^ and §44 establishes the right to view one’s personal data that is stored by the government.^5^ In this restricted context of Privacy, defending Privacy of Citizen’s data means ensuring security of personal data^6^ in government information systems, and providing information on how that data is processed. We also take into account the statement by the European Parliamentary Assembly that "in view of the new communication technologies which make it possible to store and use personal data, the right to control one’s own data should be added to" the definition of right to Privacy [8]. In order to achieve this, we need transparency,^7^ digital signatures and personal message^8^ encryption, to name some of the most important requirements. The last two are enablers of private transparency, where each individual can see and control only his or her data. This also requires that the rules are public, data is protected and government institutions are individually accountable for data processing and security, in order to provide better protection for the Citizens’ private data.

Historically Estonia has plenty of examples of subjugation of human rights, often highlighting the importance of Privacy in particular. The people of Estonia have experienced oppressive and suppressive societal conditions first hand, when their fundamental rights were violated by various occupying forces and governments.^9^ Those same events, also taught Estonians valuable lessons regarding the limits of Privacy protection – principally in conditions where those in positions of power have little, or no respect, for the rule of law, then the fundamental right to Privacy might not have any relevant meaning at all. As a result, Estonians take Privacy very seriously, and have maintained it as a key topic in all discussions surrounding the development of Estonian e-government services, along with discussions involving economic stability, resource maximization and the improvement and preservation of quality of life for Estonian Citizens. These discussions have led to the realization that computerization and administration processes automation are strategic needs for the Estonian Government. These enable the provision of the same type and range of services that other, larger and wealthier states provide to their Citizens, but, to also achieve this with significantly less resources, including manpower. Such efforts and drive toward effectiveness and efficiency have enabled the Estonian e-society to flourish.

However, computer network attacks on the Estonian e-society infrastructure in 2007, not only fundamentally changed both domestic and international discussions about cyber security and Privacy, more fundamentally, but also presented great opportunities for transformative action. Developing a combination of holistic security properties and practices that are workable have been challenging, such as those that are absolutely necessary to safeguard the personal Privacy of Estonian Citizens and for all in the Estonian society.

## Estonia and the drive to e-society

Upon the rebirth of the Estonian Republic in 1991, there was a common understanding among members of the national leadership and the general public that as a newly forming State, budgets would need to be lean, if not emaciated. At the same time, there was also recognition that there was an opportunity in front of all to systematize and to institute an efficient government – from a “clean slate,” avoiding high administrative day-to-day overhead, while lowering long-term expenditures.

Estonia is situated in a very competitive environment; its neighbors are Nordic countries with a high standard and quality of living. Regionally, and in a post USSR world, a socio-economic or a governmental failure in Estonia would have also meant the possibility of setting off cascading failures elsewhere, in other newly independent states. To achieve success at the core, one of the first challenges Estonia had to confront was to determine how to innovate in the area of state administration. Digitalization of state administration was considered to be an essential mean, by which Estonia could raise the effectiveness of its government and its processes in a timely manner. A persistent human resources deficiency - for all types of standards, and specialized employment purposes, also became a motivating factor toward digitization and the mastery of safe and secure automation.

However, before any digitization of known governmental institutions and related processes could effectively take place, the Estonian government had to consider how to establish, and to maintain trust, in and about, the proposed government information systems. The core proposition was that every Citizen should have the confidence to entrust their private data to their government, and the basis for that confidence was to be the reliable means of assuring Citizens that their privacy would be protected within all government information systems. The government also had to consider the engineering demands of the systems - to enable Citizens to faultlessly rely on these systems. Lastly, the government had to make certain that information systems would not be misused against the well-being of Estonian Citizens and others in Estonian society. The Estonian government had an intimate understanding that failure to maintain privacy would largely amount to the suppression in the forecasted rates and scales of usage of automated solutions that were being planned to be implemented. An important nearby argument in consideration was that, if the necessary systems configurations (to satisfy Privacy and security etc.) were not achievable to enable proper functionality, then the government would have been straddled with the additional requirement to maintain paper based transactional systems and processes alongside automated systems, and the aims for planned economic vitalization could not have been realized.

In the here, and now, the digitalization of Estonian society is quite advanced. As an example, it is now possible to arrange almost all administrative affairs with the state on-line. There are still three known transactions that cannot be completed on-line. They are:

1. getting ID documents issued
2. getting married
3. selling real estate

Estonian government information systems currently consist [21] of 642 information systems in total, offering 4196 services,^10^ with 75,579 data objects, 12,133 personal data objects, which include 2384 delicate data objects.^11^

## Critical foundations of the Estonian e-government

Laying the foundations for e-government began even before Estonia regained her independence. The Estonian Institute of Cybernetics’ Director, Ülo Jaaksoo and Institute researcher Monika Oit had concluded that maintaining security of information is a strategic necessity for the re-emerged Republic. A research group was thus formed at the Institute, to gather the information security knowledge necessary to achieve this goal. The very first Estonian information security specialists group, which included the lead author, convened under the auspices of the Institute, where it was also determined that three essential pillars were necessary to erect and maintain a durable e-society.

**The first pillar** of effective e-government was determined to be the implementation of a unique mechanism for identification of all the Citizens of the Republic, and to have all associated objects that also needed to be qualified and quantified. Using the example of neighboring Nordic countries, Estonia launched a system that was to be based on an **identity code,** in conjunction with the Estonian population registry, which would then serve as a single authoritative information source for all government systems.^12^

**The second pillar** was to establish the means by which Citizens would be able to relate themselves with repositories and services, while preserving the necessary degree of confidentiality needed at each step, in communicating with any and all Estonian government systems. Authentication, digital signature and personalized encryption were to be provided by way of an electronic ID card. These vital mechanisms were to ensure that none could plausibly make false claims about the will, or participation of Estonian Citizens in government processes. If ever a dispute were to occur, a specific process owner would need to present durable proof of Citizen participation within said process. As an Estonian digital signature (in the way it was structured to be created) could only be created by an ID-card carrying Citizen, no one else would be able to launch, or maintain any false claims due to the involvement of a false signature origination.

Unique digital signatures were to be used with the **Estonian national ID cards** to ensure a strong tie between the content(s) of a certain data, and the individual to whom that data verifiably belonged; also it would be assured that no intermediary could surreptitiously change any data. In this way the owner of the digital signature (the original signer), is always able to control one’s own content under that signature. In comparison, it was determined that the value and security of hand-written signatures were practically nil. The highly tamper-resistant property of digital signatures was determined to be unprecedented. Additionally, the Estonian Republic’s national ID card would also enable strong messaging encryption, which only the relevant cardholder would be able to decrypt. Such an approach presented for the very first time, the means for any individual to take control of his/her data, and that the cardholder could largely ‘decouple’ data control from any institutional control.

**The third pillar** or essential attribute to an effective, efficient and timely government administration system was to be the installation of a system which would permit the wise/meaningful utility of data. There was a need for individual and societal data to be used wisely/meaningfully; for the society-wide use of data to be responsibly and cleverly engineered, developed, and installed. Such a system would not only allow the best first time use of data, but, would also support and facilitate all subsequent uses of data, and use cases for data usage. This type of installation was also needed to assist in minimizing the amount of data that would need to be stored; as the desire was to not have data blocks duplicated, strewn-about and stored all over Estonian government. Such information systems design, development, and deployment eliminated the need for massive data stores, repetitive or duplicative data-entries and reconciliation workloads, duplicated data protection demands/expenditures/schema and responsibilities. Additionally, proper engineering was to have minimized the need for storage or transmission of data, thereby eliminating or mitigating the prospect for certain types of breaches from occurring. The designed system was named “X-Road,” and it defined the baseline security standard for all data transport within the Estonian government information enterprise, enabling a more efficient and secure way to govern.

Effective and efficient governance requires the identification and qualification of information, key informational properties, and the relationships between those informational properties and Estonian Citizens, Estonian organizations and the Estonian State. For example, a registry that shows the relationship between a specific plot of land and its owner(s); a registry of people who are qualified and are licensed to practice medicine, etc. While some registries contain information that is generated by the government directly (for example, criminal records), others require the Citizens to keep it up to date (for example, the official contact information provided by the Citizen to the government). This pre-supposes the existence of a certain level of trust - between the Citizen and the State. In order to have the required amount of functionality in information systems, firstly, a level of Citizen trust in the government’s intentions and operational directions to not misuse the information (including violations of privacy) it was to collect, was sought. Secondly, a similar level of trust would need to be extended by the government to the Citizen, to provide correct information. By this mutual commitment and extension, both parties were in essence conferring trust upon the Government’s information systems to be safe and secure, to the extent of a common understanding that the personal data in the Estonian government’s information enterprise will not be accessed or modified by unauthorized parties. If this ring of trust was to have been incomplete, the government will have been unable to provide services effectively, as it could not have relied on the information in government registries, and thereby, the government will have been left unable to make educated decisions based on the information housed in government systems.

Another principle, the Estonian government needed to adopt was one directed to minimize the collection of data. Within the context of this paper, this principle should be interpreted to mean that the government should not be in a position to ever ask the Citizen for the same data-set, multiple times. For example, asking for the person’s address in relation to that person’s income tax declaration form, and to again ask for the same information in relation to a driver’s license application, etc., is a matter of high redundancy and high labor.

If any Citizen is uniquely identifiable, then, the interpretation is simply that, a Citizen address can be retrieved from existing records, and pre-filled into any form in question, for the sake of convenience and efficiency. On the other hand, if the recorded address no longer matches the reality, then the Citizen could offer a correction to it. Avoiding task and data duplication, and other forms of organizational and process inefficiencies were prime motivational drivers for Estonian e-society. These e-society desirables led to three important and necessary ingredients for effective e-governance: (1) there needed to be a way of uniquely identifying a Citizen (for example, a unique ID code), (2) there needed to be a way of authenticating (proving the identity) said Citizen in an on-line (for example, with a smart card that is issued by a trusted source) virtual world, and (3) there needed to be a way to determine the ‘master source’ for each Citizen record. It followed from (3), that some options for greater efficiency were to not keep many competing registries that had duplicate contents, but rather to consolidate registries, or to only keep unique data fields in every registry, and to query for the rest – on an as needed basis.

On the one hand, such considerations presented opportunities to provide savings associated with computational resources (for example, in data storage) and to make the national registries easier to defend - due to the reduced exposure of attack surfaces. On the other hand, such an approach could potentially have introduced a ‘single-point-of-failure’ in enterprise operations, if information systems security was not properly addressed in the design. For small States like Estonia, that routinely experience resource constraints, reasonable options are few, and far between, particularly due to the fact that Estonia does not have a large man-power base to intensively defend a multitude of competing systems. Therefore, the choice was between guarding one or a small number of qualified point(s) very well, and guarding multiple points very poorly, against accidents, malicious and non-malicious activity, against insiders - as well as outside actors.

Yet, once again, such actions tend to concentrate and aggregate the weakness in and of the system, to the center. And consequently, instead of lending focus to the protection of data, concerns and focus would have been needed to clear, and to secure, the few critical individuals who would build, and then continually guard the State’s information systems. The background and activities of all critical personnel are extensively screened, re-checked and guarded, where there is less likelihood of system misuse. While any classical records clerk could conceivably access physical records without leaving a trace, a computerized information system can be designed to automatically log every event or activity of interest, and furthermore digitally time-stamp the log to make sure that any modifications of it can be identified.

Another security deterrent present is well embedded into the approach taken in the way the Citizen portal in Estonia has been designed and deployed. It allows the Citizen to query who has accessed his/her records. If Citizens identify someone as having gained access to their personal records – when they have no authorized need to have such access, Citizens can report such invasion of Personal Privacy and Data Security, and all matters associated with such invasions will be investigated. Such a mechanism provides for the presence of another layer of detection capabilities, beyond one that is generally provided by internal systems monitoring. In Estonia, this feature has led to some very public cases of government officials being caught accessing private data of Citizens - without any legitimate and authorized reason for such access.

It is not enough to protect data at rest in government information systems. Government must also protect data in transit - from the user to the government enterprise systems, as well as all data that will be processed. Therefore, the various government information systems and components need to be securely integrated, so that the data is protected throughout its ‘life cycle,’ which includes transport/transit. Affording such protections therefore, is only partially related to the implementation of the correct technologies, and in a correct manner. Training the user base (Citizens and government officials) and establishing secure processes and procedures are equally important. The latter includes establishing clear responsibilities for the various government institutions and enforcing the agreed rules.

Unlike human beings, information system behavior is expected to be consistent and more standardized, where system inspection and audits enable the possibility to correct undesired outcomes such as discrimination, or personal privacy violations. However, Privacy advocates have argued that there is an inherent problem in the issuance of a unique personal identifier, necessary for the foundational systematization and automation of data processing for all of the people in Estonian society, as it could potentially inspire a government to use such systems to inappropriately control the population. The Estonian ID number is often compared to US Social Security Number (SSN) and the UK National Insurance (NI) number in this discussion. However, the US SSN and UK NI have been used for authentication without proof of identity [17, 10], whereas the Estonian ID number has avoided this pitfall, as it cannot be used to claim any benefits or access. By limiting identification number usage only to uniquely identifying persons (including those with identical names) in IT systems, we eliminate the problems that are well known in US and UK.

Lastly, to enable proper information system development, its deployment and a systemic review, the key principles and processes had to be codified within the Public Information Act. The system processes were to then be maintained by the Estonian Information System Authority [19], and supported by a meta-information registry, called RIHA (Riigi Infosüsteemi Haldussüsteem), or the Administration System of the State Information System [21]. Privacy of all Citizen data, and its use, was to be guarded by the Data Inspectorate. “Information processing” without a firm legal basis was, and still is, forbidden. No government institution can build any new information systems without the explicit representation of its intended purpose, and such purpose is required to be well grounded in Estonian law. This restriction was meant to explicitly prevent any part of Estonian government sidestepping well established government purposes as prescribed by law.

## Cyber attacks on Estonia in 2007

Following the turn of the twenty-first century, Russia once again started to exert its power, primarily in neighboring states that had once been ‘subject’ to the Soviet Union. This has taken various forms, ranging from economic pressure to military operations, like those against Georgia and Ukraine. Estonia’s ‘turn’ came in 2007, when tensions sparked over a memorial statue in Tallinn, which featured an Anonymous Soldier, in Soviet uniform. The statue had become the focal point of social tensions in Estonia in the preceding years. On one side - as a symbol of occupation and oppression for the majority of Estonians, and on the other side, it was considered to be a symbol of victory for the mostly Russian-speaking minority. As protests and counter-protests at the site and online became more heated in the years leading up to 2007, the Estonian Government decided to relocate the statue from a prominent location in the City Center of Tallinn (the capital) to a military cemetery, a few miles away. The decision was followed by violent riots and looting on the evening of April the 26th (the day the statue relocation process began) in Tallinn. While crowd-control on the streets was restored within hours, the event did spill over to cyberspace. Several waves of cyber attacks targeted Estonian government and private sector^13^ computer systems between April 27th and May 18th. [16].

Russian-language discussion boards and forums were used to disseminate target lists and attack tools to anyone who wanted to pitch in. The first computer network attack [16, 4] targets were Estonian government websites, which were defended by the national Computer Emergency Response Team [22]. However, attacks against other Estonian assets on the Internet followed, in the form of website defacements, viruses being sent to key government officials, overloading email inboxes, overloading telephone lines and, most visibly of all, overloading various web servers. Most of these attacks were rather simplistic and easy to defend against, but the scale at which all such activities were occurring, soon became problematic for the State. Many of the attacks were camouflaged as ‘popular protest;’ however, the real problem was not from any authentic individual online protesters. Instead, the serious attacks were caused by well-coordinated attack launch pads, known as botnets.^14^ Remarkably, Distributed Denial of Service (DDoS) attacks were preceded by some technical efforts to measure the capabilities of the targeted systems, presumably in order to increase attack efficacy. Such actions demonstrated that at least some of the attackers were well prepared for such a campaign.

At the time, it was determined that these attacks presented a high potential for damage, even if their actual effect was limited due to defensive actions taken by Estonia and her allies. E-society architects and government personnel were worried that if a massive data loss were to have occurred, such a compromise could have made the Estonian population an easy target for foreign intelligence services, and would have ultimately eroded away all trust in State information systems. The attacks damaged publicly available systems – mostly Government and private sector websites. However, the data interchange layer was not penetrated, and the effect was generally limited to temporary service outages, and much slower services in general.

In 2007, the Estonian e-society was already relatively advanced, but paper based transactional processes were still in place, and served to mitigate some of the effects in society. The finance sector had been the champion of digitalization in the Estonian society. By 2007, the banking and finance sector was so dependent on computer systems and electronic networking, that there was no hope of replacing potentially lost transaction processing throughput with manual paper based processing in any meaningful scale. The overall dependence on computer networks in Estonia was already at such a level that an otherwise penetrating and effective attack would have been devastating. This fact, and the events of April and May of 2007 drove home the message that cyber security is a most critical requirement for Estonian e-society in general, and for data privacy in particular.

## Lessons identified from the 2007 attacks

To allay fears related to the new automation technology processing and managing data, which was to be foundational to government efficiency, everyone involved realized that there had to be a way to make data reliable, available, and correct within Estonian information systems. There was a common recognition that Citizens must trust the data, and be able to make decisions based on available data, otherwise they would not only begin to question the reliability of the data source, but would ultimately fail to use those technology instruments, that were going to create a new and efficient Estonian government.

Estonia has had its share of public discussions -- on whether or not, new technologies should be adopted. In public discussions, Estonian officials fully expected that extreme personal interests and fears, whether naturally resident or otherwise stoked - could very well surface and override rational discussions. People fear the loss of personal control when there is a looming prospect of being detached from certain places, things, people or processes, to which, a personal tie has existed. Respectively, there is a tendency to argue that a new technology, when it is first deployed, is not secure. The counterargument to that is, there is no ‘secure’ version of the world to which anything can be compared. Furthermore, general arguments are frequently presented with no real assessment of the *status quo* level of security; giving insensibility every reason to persist. Security - as such, is an idealistic concept, that can neither be proven fully, nor defined universally.

The negative effect that surfaces from the aforementioned fear and insensibility is that they are often based on “unknowns” (the changes in technology, the effects of technology, etc.), which impedes the need to have strong community dialog at the national level regarding the various aspects of security.

The cyber attack on Estonian national information infrastructure had revealed a ‘digital fault line’ in the way systems were organized and made operational. The cyber attacks served to tear down existing institutional and operational trust. Furthermore, it was realized that the Estonian e-society reliance on these systems was so remarkably high, that any possible return to operational methods and means involving ‘old fashioned paper,’ and ‘sneaker-nets’ was next to impossible.

In order to maintain forward trust, the Government of Estonia initiated dialogue with Estonians, explaining to the population - what had transpired, and how the society could proceed forward, given the circumstances, and lessons learned. To achieve desired milestones and optimal results, the Estonian Government chose to not classify, or to be secretive in discussing the details surrounding the attack on its national infrastructure. The population, therefore, saw that pertinent problems were being acknowledged, and that the government was taking the proper steps to manage, and to shape a way forward beyond the first-ever cyber confrontation on a national scale. The news of Estonian infrastructure attacks had spread quickly around the world. Any attempt to conceal events surrounding the events would have been futile anyway, and would have only served to further erode trust in the Government. Further, any attempt to conceal aspects of the attack would also have further exacerbated the challenge the Government already faced to explain the nature and the extent of the attack, and the impact of the attacks on every Estonian Citizen’s personal data Privacy.

In addition, the most important issue for the Estonian population regarding the cyber attacks was the safety of their personal data resident within the Estonian State information systems, while systems were under attack. The biggest visible impact caused by the DDoS attacks was that the Estonian population temporarily lost their ability to *access* their personal information during the attack. **The 2007 attack did not** ***compromise*** **Estonian Citizen’s personal data, or privacy**. Another key point as it related to the cyber attack was that a key ‘e-society’ component was the Estonian ID card, which uses a chip and pin. Since Estonian Citizens carry their ID cards in their wallets, and had physical control over it, they were (rightly) convinced that they were still in control of the ‘keys’ to their personal data.

Estonia’s **decision to go public** on the matter of having suffered a cyber attack proved to be one of the most important of government decisions made during the time of the incident. The 2007 Estonian cyber attacks became the benchmark by which the world would thereafter gauge other cyber attacks. The Estonian experience became the backdrop for many international discussions related to information security, and personal privacy in association with computer systems.

For Estonia, the attacks created a clear understanding among the population that, given the many vehicles of e-society**, the Government is responsible for information infrastructure and its security**. Moreover, the people of Estonia expect the protection of their data in cyberspace, and that the available institutions were not sufficient to provide, or ensure, future levels of security or safety. Plans were needed to meet these expectations, and consequently, a Cybersecurity Strategy document was created in 2008 [13]. The strategy development workgroup involved government officials and private sector specialists, including the lead author. The strategic framework called for a national cyber security coordinator, and other supervisory positions in the Estonian government. The Government Information System Development Center was reorganized into an entity to be called the Estonian Information System Authority [3] (EISA).

## Lessons identified in Estonian information system authority

The biggest challenge EISA encountered in executing its duties was the need to create a cycle of learning - from mistakes that were made by all the government entities that EISA was newly responsible for, wherein, people could communicate errors and correct their behaviors. The decision makers in other organizations had to understand the need to talk openly about problems across different institutions. The required degree of openness, and the requirement to openly communicate problems across multiple institutions proved to be a deep-rooted challenge. The challenge was further complicated by the constraints in the operating environment and the fragmentation of the applicable legislation and regulations. More than ten laws had to be changed to define EISA supervisory rights. Now, an effort is undertaken to consolidate the relevant sections of law and regulation.

**Data security management is a strategic function**, because data is typically needed to manage the main processes of an organization or institution. This data often does not belong to you. It is often owned by individuals and other organizations, who have entrusted it to you, so you can do the work. When you fail in managing (incl. Protecting) the data, then people will stop sharing it with you. Therefore data security is a strategic function and it should be the responsibility of the head of institution. This responsibility cannot be delegated to the IT department. In the Estonian government institutions this was solved by a government directive specifying that the information security responsibility lies with each institution’s top level management.

**Cooperation between institutions is crucial**. The administrative processes are traversing many government institutions. For example: process of issuing driver’s license involves driver health check. The issuing institution should somehow get the the health status from medical institution. Traditional possibility is to issue health certificate and use the person to transfer data. In modern days it is waste to use people in the function of data network, as computer networks enable much better service processes.

However, the information link creates data dependence in the process, the institutions are depending on each other for data, they share the data and if any institution spoils the trust or value of this data, then everybody will lose. Spectacular data breaches will erode the trust, leading the Citizens to feel that Government as a whole is disrespecting their privacy. And the whole Government, all its institutions will lose in this case. It is in best interest of any institution to help their neighbors not to fail in data protection.

If some institutions are narrowly focused on just their individual goals, then data sharing will suffer. Processes will become suboptimal again, which harms the time savings of the Citizen and will destroy the reason to trust the Government with data. This way the whole Government ecosystem can fall apart. Therefore Government wide information security system and supervision is needed.

**The Government IT architecture process** 15 was restarted in EISA [12], in order to enforce common policy and guarantee data security in information systems across organizations. It was determined that the technological choices had been mostly correct ones, but the organizational use of this technology needed improvement.

By now the Estonian Government has a well-established requirement management system – new norms setting requirements to processes are constantly developed. This process runs quite smoothly and strives to involve all stakeholders. Estonia has also quite good technical frameworks and understanding of data stored in different institutions. And still those changes agreed with everyone tend to become unexpectedly expensive to implement, miss the requirements or funding in dependent institutions and become destructive towards shared environments.

The leadership of EISA promoted the idea of communication between managers of institutions. While the management talks only to their own IT staff, the useful knowledge in other parts of Government is not accessed. Therefore EISA organized horizontal discussion of specialists, grouped them by shared platforms and moderated discussion to enable quick identification of problems. Speed is the key here – quick discovery of requirements, which cannot be implemented because of some problems in other parts of the system, will minimize erroneous development on basis of those faulty requirements.

Our task was to create a repeatable process of *communication between the layers and across institutions*, to build supervision and to ensure appropriate handling of deviations. The specialists dealing with different technology layers have diverging languages, values and goals. Communication between them is rare. In some cases, general understanding of the other specialty is nearly impossible. Thus the specialist communication networks have to be maintained separately and regular opportunities for interlayer communication had to be created. Also a new component – data management – had to be introduced to the traditional control functions of budget, security and norms.

EISA managed to convince the government and private sector critical infrastructure owners about the need for change and for sharing information. EISA also understood that the information sharing needs to be a two-way process. If the critical infrastructure owners do not get anything meaningful back from the government, they will only report the minimal required information. However, if they see that the government provides useful analysis back, covering the entire sector, they are more likely to voluntarily share information that is relevant to other parties as well.

**The change of mind-set** demanded new norms, education (including exercises), growing social knowledge and responsibility, as well as starting regular penetration tests to measure the progress. Exercises helped to show why data security and privacy are important for each individual institution and how failures will affect strategic processes and neighboring institutions. Social responsibility restricts responsibility transfer – even if a decision maker will not suffer legal consequences of his/her choices, then social responsibility will remain personal – in a small society like Estonia, everybody will know the name of the person whose ignorance or carelessness caused a data leak. Penetration tests are technical; they give some measurement of the system security but are not understandable for the top management. In here risk maps work very effectively as a translation mechanism – they enable to say in business process terms, what a specific technical vulnerability means for the organization.

The next challenge was **exporting the data centric shared environment thinking to our international partners**. The calculation was that it would be better to let other countries use our data on (authenticated and authorized) demand, then we could limit the amount of data transferred and stored. In this case we also could provide information to Citizens, who have been accessing their data. This work resulted in Finland starting to implement X-Road [19]; UK founding D5 [11]; and the Network Information Security Directive [7] extending incident reporting obligation in EU to critical infrastructure sectors beyond telecommunications.

There is an ongoing political battle related to cryptography, where Estonian e-government designs assume that the evolution of current **networked society is only possible because of encryption**. Its impact and importance is much stronger in other sectors than national security. A state of the art - official report is issued each year on crypto algorithms [20]. It does not repeat other reports, rather, states what changes on the field of cryptography mean for information systems in Estonia. Also European Network Information Security Agency issued official report [9] on state of the art and future outlook of most used crypto algorithms security. Encryption is empowering individuals against institutions. The privacy loss happened because consumer data collection companies made collecting and selling data a business. Encryption could and should be used to restore the balance.

EISA also started to implement *blockchain technology* [27] in the government [18, 23]. Estonia had already been using hash chaining, which is a rudimentary predecessor of blockchain. We implemented blockchain technology for two purposes. First to provide proof of database record integrity. As an example, the full texts of our laws are put into blockchain, so that the integrity of it can be easily verified. The other use was tamper proofing of activity logs. The correctness of processes in IT systems demand trusting people running the system and this trust is usually provided with corroboration of other people. Blockchain enables us to prove that system configuration and activity logs are not manipulated after the agreed correct state. Replacing subjective claims with proofs provides better control over personal information processing.

Next to these successes, recent Estonian Privacy history has also seen its fair share of **failures and controversies**. One such example is the sacrifice of a nation-wide message encryption capability in favor of protecting a person’s birth date. The system would have allowed easy-to-use strong encryption for all carriers of the Estonian National ID-card, thus protecting the contents of their e-mail from the prying eyes of third parties. However, the system required a query to the database that matches the addressee’s name to her ID-code (which contains the birth date), allowing the correct addressee to be chosen among multiple people with the same name. The Estonian Chancellor of Justice decided in 2006, this query cannot be allowed, thus forfeiting any potential privacy gains from enhanced message secrecy. This lack of habitual, and relatively easy to use encryption - became even more troubling - when services started to move to the cloud.

## Healthcare information processing in Estonia

As part of an ‘e-society,’ the goal of standing-up an Estonian eHealth system was to propagate **effectiveness and efficiency in healthcare process through the proper** 16 **reuse of data**. Estonia has an aging population like most developed countries, demand for healthcare is exceeding the capacity and the capability to supply; and this gap will widen in time. As stated in the Third Pillar above, data reuse represented the prospect to suppress any procedure duplication in matters such as medical imaging and laboratory activities, further enabling the co-operation of doctors (general practitioners and specialist), and transparency into patient care. The underlying agreements, standards and centralizing of systems have been undertaken by the Estonian eHealth Foundation^17^ (EHF).

As mentioned in the introduction, not all Estonian databases are connected, or are usable in the same exact way. One example is the “Gene Bank,” which is not connected to any Estonian government health system. Its aim is to create the foundational basis for a highly effective personalized medicine construct. Genetic samples and other information are donated to the Gene Bank on a voluntary basis, which now covers 5% of the Estonian population [5].

The core of the Estonian eHealth System is the Digital Health Record system, using HL7 and DICOM message formats for interconnection. Data transport and security layer is provided for by the government’s “X-Road” middleware software.

Patient view of all healthcare data is made possible through the Estonian eHealth Patient Portal, which requires the Estonian National ID card for patient authentication, and their signature. Within the portal, a patient can assess any and all authorizations regarding her data access. By default medical specialists can access data, but **any patient can choose to deny access to any case related data**, to any, or all care providers; including one’s own general practitioner/family physician. Certain others are authorized explicitly, as having the right to review medical records or for the collection of medicines from pharmacies. Still, these parties have to be explicitly authorized by the patient, within the eHealth System. All data access within the system is recorded and can be represented for the patient, when requested.

All those who are involved in providing healthcare, such as hospitals, pharmacies, laboratories etc., are all integrated into the Estonian eHealth system. Other subsystems of eHealth are: Digital Registration, ePrescription, eLaboratory, and eCertificates. ePrescription is readily the most used service in the whole eGovernment. ePrescription’s implementation took just six months; including the complete change-over of all information systems in hospitals, general practitioners and in pharmacies.

Historically, a highly important topic related to the eHealth transformation in Estonia has been the topic of granting access to health data for research purposes. There is only a binary option in this regard for the patient -- either to allow, or to deny access to the medical data for research purposes. Anonymization or obfuscation is a routine part of granting access to medical data for research purposes. However, it is well known that anonymization or obfuscation offer only weak protections. Patients, however, desire more integrated safety and control over their data and if binary choices are the only options presented, patient decisions tend to lean towards denying access to medical data for research purposes. EHF is searching for a solution that can be implemented to empower all Estonian patients to implement a system of continuous consent, where patient can routinely decide, for which reason or circumstance he permits data access, and under which circumstances he withdraws such consent.

Informational control associated with Medical research poses the biggest strain upon Privacy, and searching for a workable personal data control model will likely be the most intense endeavor of all. If working solutions are found, then they can be applied to most, if not all of the data stores in eGovernments as well as other domains.

## Forward privacy implications

Healthcare industry has become data dependent. Information systems are used to raise the productivity of medical workers. With constrained budgets, aging population and rising service level expectations, there is no option to simply switch the systems off. We can say hospitals are in a similar situation, where Estonia found itself, in 2007. Personal health information is strategically needed in the industry, and therefore the **data protection has to be “the” top priority**.

Significant efforts must also be undertaken to ensure that the volume of data in use can be minimized to make certain that data protection is realistically possible. And as paradoxical as it might appear, effective data protection also needs to be complemented with processes that ensure effective data sharing. To accomplish such tasks, trust in healthcare ecosystems must exist, so patients could have confidence in the manner that medical professionals will handle their data. In order to generate trust, we must:

1. Build transparent processes that give the meta information and control over data to the patient. Then we can also negotiate processing exceptions on a case by case basis.
2. Exchange claims for proofs. Use modern technology to provide the proof.
3. Build data centric protection. It means using encryption to define defense boundaries and also using data in a process agnostic way, agreeing the formats and mapping, leaving the concrete process decisions to healthcare professionals to the limit that the processes are compatible with agreed data formats or can be mapped to an agreed format.
4. Create information security cooperation with healthcare professionals.

**Infrastructure** should be also developed to support the ecosystem. Secure data exchange platforms are needed along with corresponding baseline standards. Identity management and strong authentication are needed for building strong relations with all patients, in order to increase efficiency and patient trust in the ecosystem. Health insurance companies or institutions that finance a large part of the industry should be interested in a trustworthy infrastructure and should put their effort into making the corresponding, and necessary changes.

An emerging idea is the idea of a **personal data market**. It is quite clear that perceived value of privacy and the understanding of various risks and implications vary widely among people. Making general rules universally applicable to everybody would not be acceptable to most. One possible solution is to organize the enterprise data market, where companies and researchers are able to present data use and licensing/rental/sales related propositions, assign pricing models, where patients are then able to choose to either license, rent, sell or to withdraw their data from use. A market model for ‘personal data,’ will help to map essential inputs with respect to the value people place on Privacy vs. Money. Within such a market, self-regulating behavior has the prospect to sprout. Furthermore, details that emerge from qualifying and quantifying market behaviors can prospectively be utilized as a basis for engineering better health information systems with adequate defenses, modeled according to value placements made.

## Conclusions

Estonia has in its history, a fair share of events demonstrating the value of human rights in general and privacy in particular. The same events also taught lessons on the limits of privacy protection – when leaders of nations express little or no respect for laws and human rights, then privacy is likely to establish, neither a reasonable, nor a firm meaning in society. However, a law abiding society can benefit greatly by using information systems to provide both greater efficiency and greater control of personal data, while initiating the necessary foundations for a functioning e-society.

Effective and efficient e-governance - require the populations to trust government information systems. If this trust is lacking, then Citizens simply would refuse to give their personal data to be processed by government systems, and the intended gains in efficient and effective administration and governance would be lost. Measures to build trust include transparency, digital signatures and personal message encryption. Such measures will provide Citizens with more control over their private data in government information systems. In Estonia, the ID card is the Citizen’s key to government information systems, allowing her to monitor who has accessed her data and, in the case of personal medical information, authorize or deny such access at the level of individual doctors. This enforces the Estonian Citizen’s ownership of, and unprecedented control over, her Private data.

Being among the early adopters of digital health care solutions, Estonia has had plenty of time to discuss and learn from her experiences. A key lesson from these discussions is the need for security as a precondition of maintaining data Privacy. While Privacy discussions often revolve around confidentiality, we argue that integrity and the availability of Private data are equally necessary aspects of Privacy protection. There is no point in asking Citizens for their private information, if it cannot be trusted to be ‘true’, or if it cannot be accessed for legitimate purposes it was collected for, in the first instance. Therefore government institutions must strive to ensure the confidentiality, integrity and availability of all personal data - entrusted to them.

## Footnotes

1 Much of the insights and conclusions presented herein stem from the personal experience of the lead author, who was directly involved in designing the technical foundations of the Estonian e-society, and also served as the founding Director General of the Estonian Information System Authority (EISA). At EISA, the lead author faced the challenge to establish an organization, and a set of practices, to secure government information systems - in a more systematic way, as never before. The government information systems were already integrated so tightly that it started to be considered as one system and EISA was tasked to build integrated governance and defense. This was also one reason behind having a singular ‘System’ in the name of the organization that oversees the multitude of government information systems.

2 Often used interchangeably, e-safety and e-security are addressed here as someone’s personal choices and behaviors online (e-safety) and service providers’ measures to assure the user with a secure online experience (e-security).

3 In the commented version of the Constitution of Estonia [1]: §26 is shortly explained as personal privacy and the privacy of family life among others as personal autonomy, identity, personal immunity, privacy, personal development, identification and other qualities of one’s personality. Only in Estonian.

4 In the commented version of the Constitution of Estonia [1] §43 message secrecy applies to messages that are sent using general service means. General service is communication service that is provided by communication companies for the public to use. The Constitution of Estonia protects also private communication, that does not use public means (eg private conversation), but Estonian Supreme Court has ruled that private communication is protected by Constitution §26 that concerns private life, not §43 that is explained here.

5 The Constitution states that all citizens have the right to peruse all materials gathered or held in state organizations and local authorities, also including archives of these institutions.

6 Ensuring information security means providing three basic properties: confidentiality, availability and integrity. Privacy discussion often focuses on confidentiality and equates privacy loss with a data leak. However, the other two security properties should be considered as well, since there is very little value in processing incorrect data (lack of integrity) or storing data that cannot be accessed when needed (lack of availability). If we are unable to ensure integrity and availability to a reasonable degree, then the data should not be collected in the first place and the confidentiality of absent data becomes irrelevant in the context of privacy.

7 In this document, “transparency”, is used in the context of Estonian Public Information Act https://www.riigiteataja.ee/en/eli/518012016001/consolide. The purpose of this Act is to ensure that the public and every person has the opportunity to access information intended for public use, based on the principles of a democratic and social rule of law and an open society, and to create opportunities for the public to monitor the performance of public duties.

8 Includes SMS, e-mails, instant messages etc.

9 During and after World War II Estonia was occupied three times, twice by the Soviet Union and once by Nazi Germany. Both foreign powers violated the rights of Estonian Citizens, including gross violations of their Privacy, which sometimes led to their deportation and/or death.

10 For example, the Marital property register has 5 services: find registry card (for X-road 5 & 6), electronic manual in the web, the web query service and the web xml service.

11 data object is a type of data stored in information systems, eg. date when passport was issued; personal data object is type of data, eg. identifying signs of a person who has entry ban to EU; delicate data object is type of data eg. a person’s medical diagnosis code, as in ICD-10

12 While the adopted code system is easy to remember, it does have its shortcomings. The code includes data about the birth date and sex of the person, which has caused some problems in implementation. For example, it is problematic for people who undergo sex change.

13 At the time, the lead author was the head of IT security for Hansapank, the largest banking establishment in the Baltic region. Hansapank’s web servers were among the hardest hit.

14 Botnet is a network of distributed or clustered computers controlled centrally by botmaster via Command and Control channel. Bots (“robots” or “zombies”) are essentially individual computers, which become an instrument of the Cyberattack without the knowledge of its owner.

15 Current status is reflected in architecture wiki https://www.ria.ee/riigiarhitektuur/wiki/doku.php?id=algus

16 Well defined authorities and security frameworks

17 See: [26] for more information
